# Activated carbon decreases invasive plant growth by mediating plant–microbe interactions

**DOI:** 10.1093/aobpla/plu072

**Published:** 2014-11-10

**Authors:** Nicole E. Nolan, Andrew Kulmatiski, Karen H. Beard, Jeanette M. Norton

**Affiliations:** 1 Department of Wildland Resources and the Ecology Center, Utah Agricultural Experiment Station, Utah State University, Logan, UT 84322-5230, USA; 2 Department of Plant, Soil, and Climate, Utah State University, Logan, UT 84322-4820, USA

**Keywords:** Allelopathy, Archaea, bacteria, invasive species, native plants, plant–soil feedback, pyrosequencing, restoration.

## Abstract

There is growing appreciation for the idea that plant–soil interactions (e.g. allelopathy and plant–microbe feedbacks) may explain the success of some non-native plants. Where this is the case, native plant restoration may require management tools that change plant–soil interactions. Activated carbon (AC) is one such potential tool. Previous research has shown the potential for high concentrations of AC to restore native plant growth to areas dominated by non-natives on a small scale (1 m × 1 m plots). Here we (i) test the efficacy of different AC concentrations at a larger scale (15 m × 15 m plots), (ii) measure microbial responses to AC treatment and (iii) use a greenhouse experiment to identify the primary mechanism, allelopathy versus microbial changes, through which AC impacts native and non-native plant growth. Three years after large-scale applications, AC treatments decreased non-native plant cover and increased the ratio of native to non-native species cover, particularly at concentrations >400 g m^−2^. Activated carbon similarly decreased non-native plant growth in the greenhouse. This effect, however, was only observed in live soils, suggesting that AC effects were microbially mediated and not caused by direct allelopathy. Bacterial community analysis of field soils indicated that AC increased the relative abundance of an unidentified bacterium and an Actinomycetales and decreased the relative abundance of a Flavobacterium, suggesting that these organisms may play a role in AC effects on plant growth. Results support the idea that manipulations of plant–microbe interactions may provide novel and effective ways of directing plant growth and community development (e.g. native plant restoration).

## Abstract (precis)

Research using activated carbon shows promise for a new approach to managing weeds and plant communities. Researchers find that activated carbon added to the soil can change the way plants and soil organisms communicate with each other. Changing this communication can improve native plant growth.

## Introduction

A growing body of research demonstrates that belowground processes often increase invasive plant growth relative to native plants (Callaway and Aschehoug 2000; Ehrenfeld and Scott 2001; van der Heijden *et al*. 2008; Parepa *et al*. 2013). This can occur through several processes. Invasives have been found to benefit from microbial symbiont accumulation (Yuan *et al*. 2014), belowground enemy release (Kulmatiski *et al*. 2008), accumulation of belowground pests that decrease the growth of native competitors (Eppinga *et al*. 2006; Mangla *et al*. 2008), novel weapons (i.e. allelopathy; Bais *et al.* 2003) and increased nutrient cycling rates caused by disturbance or the invasive (typically non-native) plants themselves (Ehrenfeld and Scott 2001; Hawkes *et al*. 2005).

All of these mechanisms, with the potential exception of ‘novel weapons’, develop as a result of communication between plants and soil organisms via small organic molecules (Bais *et al*. 2004). Broadly, relative to native plants, invasive plants often benefit from plant–soil communication. Thus, treatments that can decrease plant–soil communication and plant–soil feedbacks can be expected to decrease invasive plant growth.

Of course, not all invasives rely on belowground processes to succeed, but for those that do, soil-based management approaches may provide novel and necessary tools for restoring native plants and the ecosystem services they provide (Kulmatiski *et al*. 2006; Cramer *et al*. 2008; Eviner and Hawkes 2008). Unfortunately, studies that determine the importance of belowground processes relative to other plant growth factors (e.g. dispersal, herbivory, etc.) are relatively rare (Kulmatiski *et al*. 2006). More studies are needed to determine whether or not soil-based techniques can be effective in field settings.

One recently developed belowground approach that has been used to decrease invasive plant growth is the use of activated carbon (AC). Made from coal, nutshells or wood, AC is a highly porous substance that binds organic compounds through van der Waals force. Activated carbon is essentially a cleaner form of biochar from which everything except the carbon skeleton has been removed. Activated carbon adsorbs most organic compounds, but is poor at adsorbing alcohols, glycols and non-organic compounds (e.g. ammonia and nitrate; Cheremisinoff and Ellerbusch 1978). Activated carbon is therefore different from biochar in that AC is less likely to increase microbial activity or plant growth by releasing nutrients, increasing pH or releasing volatile organics (Lehmann *et al*. 2011; Biederman and Harpole 2013). Activated carbon is also different from reduced organic compounds (i.e. sugar or sawdust) in that AC does not provide resources to heterotrophic organisms and is therefore not expected to affect non-native plants by slowing nutrient cycling rates (Blumenthal *et al.* 2003; Blumenthal 2009). Rather, the primary mechanism through which AC is likely to affect plant growth is by binding organic molecules.

Activated carbon has been used extensively in allelopathy research for its ability to bind organic molecules (Callaway and Aschehoug 2000; Inderjit and Callaway 2003; Abhilasha *et al*. 2008; Barney *et al*. 2009; Jarchow and Cook 2009; Kabouw *et al*. 2010). This research suggests that AC could be used to decrease the benefits of novel weapons by non-native plants. However, by indiscriminately binding organic molecules, AC may have a greater effect on plant growth by slowing communication among soil organisms (Kulmatiski and Beard 2006; Lau *et al*. 2008; Schertzer *et al*. 2009; Wurst and Van Beersum 2009; Kulmatiski 2011; Del Fabbro *et al*. 2014). In support of this idea, Abhilasha *et al.* (2008) found that AC effects were sometimes present in live soils but not in sterilized soils, indicating that AC effects were sometimes caused by plant–microbe interactions. Further, Kulmatiski (2011) found that AC addition changed soil microbial community composition and subsequent plant growth. The relative importance of these mechanisms remains unknown but in either case (i.e. allelopathy or plant–microbe communication), AC may be expected to decrease non-native growth.

In a rare field test using AC, Kulmatiski and Beard (2006) found that high concentrations (i.e. 1000 g m^−2^) of AC decreased non-native plant growth and increased native plant growth in small field plots. That research suggested that AC effects were caused by decreasing either allelopathy or plant–microbe communication but not likely due to changes in nutrient availability (Kulmatiski 2006, 2011). The goals of the present study were 3 folds: (i) to test AC effectiveness in restoring native plant communities in large-scale treatments, (ii) to identify the lowest effective AC concentration and (iii) to determine whether AC effects on plant growth are microbially mediated or caused by influencing the effects of allelopathy. To address the first and second goals, we monitored plant composition in large (15 m × 15 m plots) plots treated with different concentrations of AC and control plots in nine non-native-plant-dominated fields. We used two approaches to address the third goal. First, we assessed how AC treatments and control plots affected soil microbial composition using pyrosequencing to describe the archaeal and bacterial communities. Second, to determine whether AC effects required living microbial communities, plant growth in live and sterilized AC-treated soils was compared in a greenhouse experiment.

## Methods

### Field experiment

To test the effectiveness of AC in the field at a large scale, an experiment was conducted in nine former agricultural fields located in the Methow Valley, WA, USA (48°37′N, 107°10′W, 580–880 m above sea level). Fields were on Newbon-Conconully soil series association (coarse-loamy, mixed mesic Typic Haploxerolls). This semi-arid shrub-steppe ecotype has a mean annual precipitation of 380 mm, primarily occurring as snow outside of the growing season (October–March).

The vegetation and management history of the study area is representative of a significant portion of the northern Intermountain West; abandoned agricultural fields are dominated by non-native plants (Sheley and Petroff 1998). Dominant non-native species include grasses: *Bromus inermis* Leyss. DC, *Bromus tectorum* L. and *Poa bulbosa* L.; and forbs: *Cardaria draba* L. Desv., *Centaurea diffusa* Lam., *Lactuca serriola* L., *Medicago sativa* L. and *Sisymbrium* spp. (*S. altissimum* L. and *S. loeselli* L.). Native vegetation areas that have never been used for agriculture surround these abandoned agricultural fields and include grasses: *Festuca idahoensis* Elmer., *Koeleria macrantha* (Ledeb.) Schult. and *Pseudoroegneria spicata* Pursh.; forbs: *Balsamorhiza sagittata* Pursh. and *Lupinus* spp. (*L. arbustus* Dougl., *L. aridis* Dougl. and *L. sericeus* Pursh.) and shrubs: *Artemisia tridentata* Nutt and *Purshia tridentata* Pursh. (Kulmatiski 2006). Species in this area can be categorized as non-native or native **[see Supporting Information]**. Native species are the ones commonly found in undisturbed native-dominated fields. Non-native species include non-native species and some native annual forbs, for example *Amsinckia menziesii*, which are found almost exclusively in non-native plant-dominated abandoned agricultural fields. Species that are considered non-native are actively managed by private and state landowners in the study area.

At each of the nine sites, seven 15 m × 15 m plots were established with a 5-m buffer between plots and the following treatments were applied randomly: four concentrations of coal-based AC, 100 g m^−2^ (100), 400 g m^−2^ (400), 700 g m^−2^ (700), 1000 g m^−2^ (1000), one wood-based AC at 1000 g m^−2^ (1000w) and a control that received the same physical disturbance and seeding as the treatments but no AC, 0 g m^−2^ (0). Each site also had one ‘complete’ control (CC) with the same physical disturbance but no AC or seed addition. Activated carbon used was a commercial grade, coal-based carbon powder with a 300 mesh size and iodine number (measure of pore content) >500 mg g^−1^ and wood-based carbon with a 330 mesh size and iodine number >500 mg g^−1^ (Carbon Activated Corporation, Compton, CA, USA).

Sites were treated in October 2010. Activated carbon was manually applied in powdered form using a push seed spreader and mixed into the top soil to 15 cm depth using two passes with a disc harrow pulled by a tractor. This soil mixing also removed standing vegetation. A mix of native seeds was broadcast by a hand seed spreader at a rate of 5.6 g m^−2^. The seed mix included by weight: 7.5 % *B. sagittata*, 12.6 % *Collomia grandiflora* Douglas ex Lindl., 17.9 % *F. idahoensis*, 17.4 % *K. macrantha*, 9.0 % *L. sericeus*, 7.9 % *Lomatium dissectum* Nutt. and 27.7 % *P. spicata*, using local varieties when available (BFI Native Seeds, Moses Lake, WA, USA).

Vegetation responses to AC treatments were assessed using the point-intercept method. Vegetation was assessed in each of nine 1-m^2^ subplots located in a 3 m × 3 m grid within each plot. Vegetation surveys were performed during the peak growing season (June) in 2011–2013. Additionally, germination success was assessed in three 10 cm by 10 cm in each of the nine subplots within each plot, in May 2011.

To determine the direct effect of AC on archaeal and bacterial communities, three soil cores (0–15 cm) were extracted from random locations from the 0 and 1000 plots and from undisturbed native-plant-dominated (native) plots that were adjacent to the study plots. Soil samples were placed on ice in the field and taken to a −20 °C freezer within 24 h. DNA from these soil samples was extracted using an Ultraclean-htp 96-well soil DNA kit (MoBio, Carlsbad, CA) from homogenized 0.5 g samples. DNA was further purified using the MinElute reaction cleanup kit (QIAGEN). DNA concentration and purity were determined using a Nanodrop ND-2000 spectrophotometer (NanoDrop, DE). Purified DNA extracts were stored at −80 °C. Amplification was performed with primers targeting the variable region V4–V5 of the 16S rRNA gene (515F and 907R) with 10 bp unique barcode on the forward primer (He *et al*. 2010). Amplicons were purified using AMPure XP beads (Agencourt, Beckman Coulter Indianapolis, IN), quantified using Quant-iT™. PicoGreen Kit (Invitrogen) and then pooled in equimolar amounts. Unidirectional pyrosequencing was performed on a GS-Flex Titanium 454 platform according to the manufacturer's directions (454 Life Sciences, Roche Applied Sciences, Branford, CT) at the USU Center for Integrated Biosystems (Fierer *et al*. 2012). The open source software package Quantitative Insights into Microbial Ecology (QIIME; Caporaso *et al.* 2010) was used to manage the analysis flow. Sequences were denoised, sorted by barcode and operational taxonomic units (OTUs) identified (97 % identity). Representative OTUs were assigned to a taxonomic identity using a reference database. The Python Nearest Alignment Space Termination tool (PyNAST) was used for multiple sequence alignment. Classification was performed to the lowest taxonomic level possible (Fierer *et al*. 2012). Some OTUs were only identifiable to kingdom while others were identifiable to genus.

In 2011, to determine the effect of AC on N cycling rates, N mineralization rates were determined in the 0, 100, 1000 and CC plots for all nine sites using the buried bag technique (Robertson *et al.* 1999). Briefly, in early June, 10, 4-cm-diameter core samples were collected to a depth of 15 cm in each plot. Five samples were processed immediately and five were placed individually into polyethylene bags and reburied for 1 month. From each core sample, 10 g of soil was extracted in 100 mL of 2.0 M KCl. Inorganic N was determined from colorimetric analysis of KCl extracts using a Lachat autoanalyser (Lachat Instruments, Loveland, CO, USA). Rates of microbial mineralization were calculated using the change in NH^+^_4_ and NO^−^_3_ [incubated (NH^+^_4_ + NO^−^_3_) − initial field (NH^+^_4_ + NO^−^_3_)] and nitrification rates were calculated from the change in NO^−^_3_ (incubated NO^−^_3_ − initial field NO^−^_3_).

### Greenhouse experiment

To isolate the mechanism through which AC changes plant growth, a greenhouse experiment was conducted in an Agricultural Research Station greenhouse facility at Utah State University, Logan, UT, USA, from August to November 2011. A total of 740 1-L polyethylene pots (7.6 cm width and 20.3 cm height; Stuewe & Sons, Model MT38, Tangent, Oregon, USA) were randomly assigned to one of three soil treatments: live soil with AC, sterile soil with AC and a control live soil. All pots were filled with a steam sterilized 6 : 1 ratio of sand and peat. The two treatments that received live soils (i.e. both AC live and control live) were inoculated with 5 % by volume field soil collected from the Methow Valley. Similar to the 1000 treatments in the field experiment, the two AC treatments received 1 % AC by mass, which was mixed throughout the pot. A coal-based, 300 mesh, commercial grade AC was used. To sterilize soils with minimal physical disruption, soils in ‘AC sterile’ treatments were inoculated with 5 % by volume gamma-irradiated (25 kGy) field soil prior to AC addition (JS8900 Batch Gamma Irradiator, Steris Isomedix, Temecula, CA, USA; van Grunsven *et al.* 2007; Berns *et al.* 2008). As with any greenhouse experiments, sterilized soils were likely to be re-colonized by soil organisms during the experiment, though sterilized soils were expected to maintain smaller and less diverse microbial communities throughout the experiment (Tanaka *et al*. 2003).

One native and one non-native plant species were grown in each pot. For the plant species listed below, all possible native–non-native pairing combinations were replicated for each treatment. Each pairing was replicated 15 times in AC-live and control-live treatments, and 12 times in AC-sterile treatments. Species included the most common species found at the study site: three native grass species: *F. idahoensis*, *K. macrantha* and *P. spicata*, and two native forbs: *L. sericeus* and *B. sagittata*, and five common non-native species: the grass *B. tectorum* and four non-native forbs: *C. diffusa*, *L. serriola*, *Tragopogon dubius* Scop. and *S. altissimum. B. sagittata* grew in <10 % of the pots and was removed from primary analysis. Non-native plant growth in the pots in which *B. sagittata* never grew was used as a measure of the direct effects of AC and sterilization on non-native plants (i.e. in the absence of competitors). Native seeds were purchased and local Methow Valley varieties were used when possible (BFI Native Seeds). Non-native seeds were collected from the Methow Valley in summer 2011.

Three successful germinants of each species were planted per pot. After 3 weeks, the tallest individual of each species was allowed to remain in each pot. Greenhouse conditions were at a mean temperature of 22 °C and sodium lamps were used to maintain 14 h of light daily. All pots were watered daily. Pots were rotated within the greenhouse weekly. After 3 months aboveground biomass and belowground biomass for each species in each pot were dried at 70 °C until constant weight, weighed and recorded. For consistency with the field experiment, we refer to native species in the greenhouse experiment as native and non-native species as non-native.

### Statistical analyses

Two sets of tests were conducted on the field data. First, to determine if results were similar to those from the previous small-scale experiment (Kulmatiski and Beard 2006), we tested the effect of the high concentration of AC on plant growth responses. Second, to determine at which concentration AC had measurable effects on plant growth, we tested the effects of multiple AC concentrations.

To test the effect of the high concentration treatment, treatment effects on the 0, 1000 and CC plots were tested on the percent cover of native species, the percent cover of non-native species and the ratio of native to non-native species (native : non-native) using a two-way randomized block design with repeated-measures and subsample analysis of variance (ANOVA). Fixed effects were treatment and year, and random effects were field and treatment within field.

To compare the effects of different AC concentrations, treatment effects in the 0, 100, 400, 700, 1000 and 1000w plots were tested on the percent cover of native species, non-native species and native : non-native ratio using a two-way randomized block design with repeated-measures and subsamples ANOVA. The fixed effect was treatment and year, and random effects were field and treatment within field. Treatment effects on germination, net N mineralization and nitrification rates were treated separately using a one-way randomized block design with repeated-measures and subsample ANOVA. For all tests, a post-hoc Tukey–Kramer method was used to adjust for Type I error and determine pairwise differences among least square means. Means from raw data are reported.

Differences in microbial community composition between 0, 1000 and native plots both early and late in the growing season were explored using principal component analyses. Operational taxonomic units not present in at least 10 % of the samples were removed from analyses because such uncommon organisms were unlikely to distinguish treatments. Operational taxonomic unit abundances determined for replicate cores within a plot were averaged prior to analyses. All analyses were performed using the Vegan package in ‘R’ (R Core Research Team 2004). Within Vegan, a Bray–Curtis distance metric was used to calculate a distance matrix from the normalized intensity values for the OTUs to perform statistical analyses of similarities (ANOSIM; R Core Research Team 2004).

For the greenhouse experiment, effects of treatment were tested on the ratio of native : non-native aboveground biomass, native aboveground biomass, non-native aboveground biomass, native belowground biomass and non-native belowground biomass using a three-way factorial in a completely randomized design ANOVA. Fixed effects were treatment, native species and non-native species; pots were considered replicates and included in residual effects. Additionally, monoculture non-native biomass was tested in a one-way factorial with treatment and non-native species as fixed effects. Post-hoc Tukey–Kramer was used to adjust for Type I error and determine pairwise differences among least-squared means. Prior to the analysis, pots with no total growth were removed from the dataset. Above- and belowground biomass raw values were square-root transformed and native : non-native aboveground ratio data were log-transformed to better meet assumptions of normality and homogeneity of variance. All tests were considered significant at the *α* = 0.05 level, marginally significant when 0.05 < *α* < 0.10. All significant differences indicated in figures are at the *α* = 0.05 level. All field and greenhouse analyses were conducted using the GLIMMIX procedure in SAS for Windows v. 9.3 (SAS Institute, Inc., Cary, NC, USA).

## Results

### Field experiment

#### Large-scale application test

**Figure 1.**
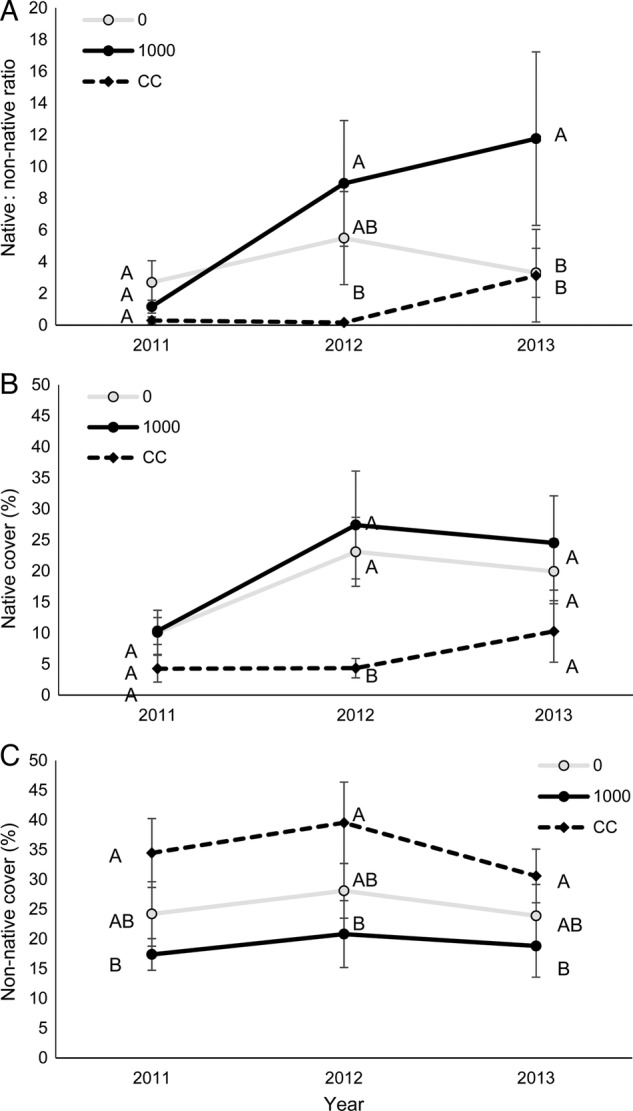
Treatments CC, 0 and 1000 g m^−2^ for each year for (A) native : non-native ratio, (B) native species percent cover and (C) non-native percent cover (mean ± SE). Letters denote differences among treatments within years at the α <0.05 level.

There was an interaction effect between treatment and year for the native : non-native ratio and for native plant cover, but not for non-native plant cover (*F*_4,696_ = 7.12, *P* < 0.01; *F*_4,696_ = 13.49, *P* < 0.01 and *F*_4,696_ = 0.93, *P* = 0.43, respectively; Fig. 1), so post-hoc comparisons (Tukey–Kramer adjusted) of treatment × year combinations were examined. While there were no differences among treatments in 2011, the native : non-native ratio was greater in 1000 treatments than in CC treatments in 2012 (*T*_1,696_ = 3.31, *P* = 0.03) and was greater than both 0 and CC treatments in 2013 (*T*_1,696_ = 3.19, *P* = 0.04 and *T*_1,696_ = 3.26, *P* = 0.03, respectively; Fig. 1A). While there was no difference among treatments in 2011, native plant cover was greater in 1000 and 0 treatments than in CC treatments in 2012 (*T*_1,696_ = 4.82, *P* < 0.01 and *T*_1,696_ = 3.92, *P* < 0.01, respectively; Fig. 1B). In 2013, the difference in native plant cover between 1000 and CC plots remained marginally significant (*T*_1,696_ = 2.98, *P* = 0.07), but native plant cover was no longer greater in 0 than CC plots (*T*_1,696_ = 2.02, *P* = 0.53). There was no treatment × year interaction for non-native cover (*F*_4,696_ = 0.93, *P* = 0.45; Fig. 1C); non-native plant cover was greater in CC than 1000 treatments across years (*F*_2,16_ = 4.58, *P* = 0.03).

#### Comparing different concentrations of AC

**Figure 2.**
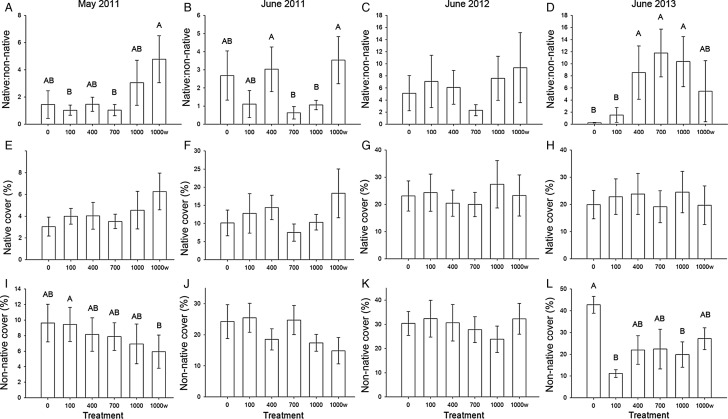
(A–L) Treatments 0, 400, 700, 1000 and 1000w g AC m^−2^ for May 2011, June 2011, June 2012 and June 2013 for native : non-native species cover ratio, native species percent cover and non-native species percent cover (mean ± SE). Letters denote significant differences between treatments at the α < 0.05 level.

In May 2011, there was a treatment effect on the native : non-native germination ratio (*F*_5,40_ = 3.01, *P*= 0.02); 1000w had a greater ratio than 100 or 700 (*T*_1,40_ = 3.02, *P* = 0.05 and *T*_1,40_ = 3.16, *P* = 0.03; Fig. 2A–D). 1000w also had a marginally greater native : non-native ratio than 0 and 400 treatments (*T*_1,40_ = 2.81, *P* = 0.08 and *T*_1,40_ = 2.80, *P* = 0.08). There was no effect of AC treatment on native species germination (*F*_5,40_ = 0.53, *P* = 0.75; Fig. 2E–H), but there was an effect of treatment on non-native species germination (*F*_5,40_ = 3.00, *P* = 0.02); 100 had greater germination than 1000w (*T*_1,40_ = 3.32, *P* = 0.02) and 700 was marginally greater than 1000w (*T*_1,40_ = 2.79, *P* = 0.08; Fig. 2I–L).

There was an interaction effect between treatment and year for peak growing season native: non-native ratio, native plant cover and non-native plant cover (*F*_10,1245_ = 6.98, *P* < 0.01; *F*_10,1288_ = 3.52, *P* < 0.01 and *F*_10,1309_ = 10.64, *P* < 0.01, respectively) so post-hoc comparisons (Tukey–Kramer adjusted) of treatment × year combinations were examined.

In June 2011, the native : non-native ratio was greater in 400 and 1000w treatments than in 700 treatments (*T*_1,1223_ = 3.51, *P* = 0.05 and *T*_2,1223_ = 3.93, *P* = 0.01; Fig. 2A) or 1000 treatments (*T*_1,1223_ = 3.51, *P* = 0.05 and *T*_1,1223_ = 3.93, *P* = 0.01). Neither native nor non-native cover differed among treatments in June 2011. In June 2012, there were no differences in the native : non-native ratio, native cover or non-native cover among treatments. In June 2013, the native : non-native ratio was greater in 400, 700 and 1000 treatments than in 0 treatments (*T*_1,1223_ = 3.64, *P* = 0.03; *T*_1,1223_ = 4.44, *P* < 0.01 and *T*_1,1223_ = 4.12, *P* < 0.01, respectively) and 100 treatments (*T*_1,1254_ = 4.48, *P* < 0.01; *T*_1,1254_ = 4.08, *P* = 0.01 and *T*_1,1254_ = 4.61, *P* < 0.01, respectively). Native cover did not differ among treatments in 2013. Non-native cover was greater in 0 treatments than 100 and 1000 treatments (*T*_1,1288_ = 3.68, *P* = 0.03 and T_1,1288_ = 3.59, *P* = 0.04, respectively).

#### Soil N and microbial responses to field treatments

There was no treatment effect for net N mineralization or nitrification rates (*F*_3,21_ = 0.37, *P* = 0.78 and *F_3_*_,21_ = 0.67, *P* = 0.58). Mean final N levels were 0.35 mg NH^+^_4_ kg^−1^ soil and 1.23 mg NO^−^_3_ kg^−1^ soil, with a mean net N mineralization rate of 0.029 mg kg^−1^ day^−1^ and a mean nitrification rate of 0.028 mg kg^−1^ day^−1^.

**Figure 3.**
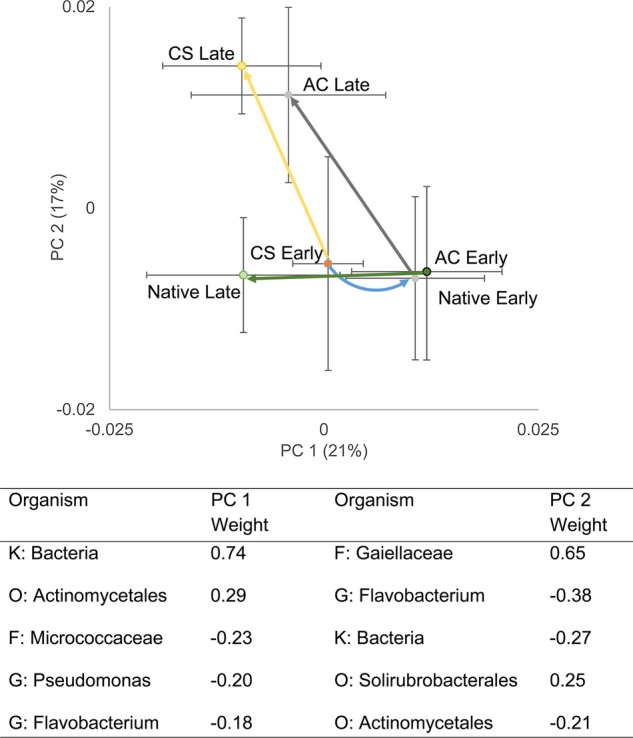
Principal component analysis of archaeal and bacterial OTUs associated with native plant communities (native), control + seed-treated soils (CS) and activated-carbon-treated (AC) soils both early in the growing season prior to plant growth (early) and near the end of the growing season (late). The blue arrow shows the effect of adding AC to treated soils. The grey, yellow and green arrows show how microbial communities changed over the growing season in AC-treated, seeded and native plots, respectively. The table inset shows the bacteria associated with PC1 and PC2. K = kingdom, F = family, O = order and G = genus.

Between early and late sampling, microbial community composition in all treatments became more negative along PC axis 1 (Fig. 3; ANOSIM statistic = 0.21, *P* = 0.01). Early in the season, AC made microbial communities similar to native soil. This was due to decreases in an unidentified bacteria and an Actinomycetales, and increases in aMicrococcaceae, a Pseudomonas and a Flavobacterium species (Fig. 2, ANOSIM statistic = 0.21, *P* = 0.01). Late in the season, native soils were distinguished from 0 and 1000 soils by an increase in the unidentified bacteria and Actinomycetales and a decrease in Flavobacterium.

### Greenhouse experiment

**Figure 4.**
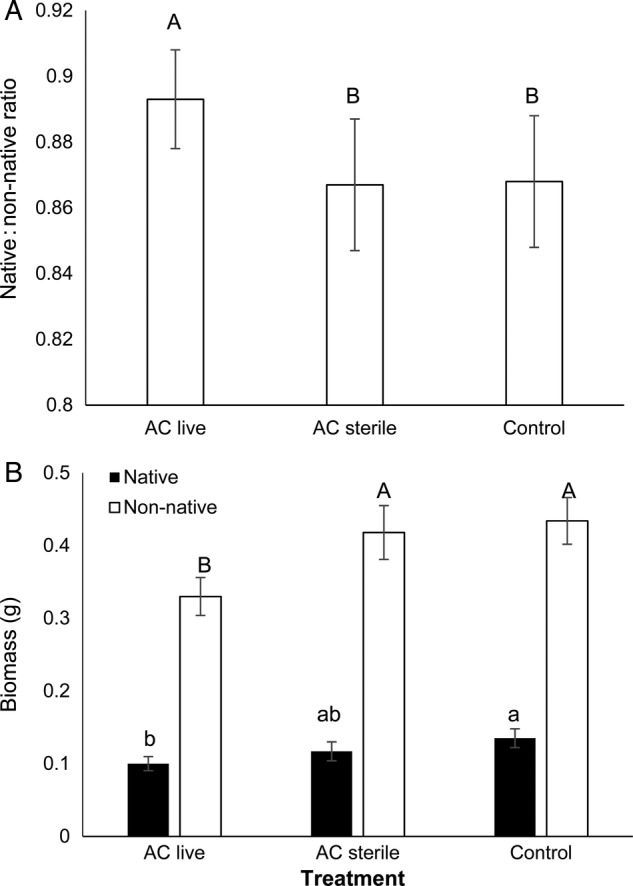
(A) Native : non-native species aboveground biomass ratio in control, activated-carbon-treated (AC live) and activated-carbon-treated and sterilized (AC sterile) soils. (B) Total aboveground biomass for all native species and non-native species in control, activated-carbon-treated (AC live) and activated-carbon-treated and sterilized (AC sterile) soils (mean ± SE). Letters denote differences among treatments at the α < 0.05 level.

**Figure 5.**
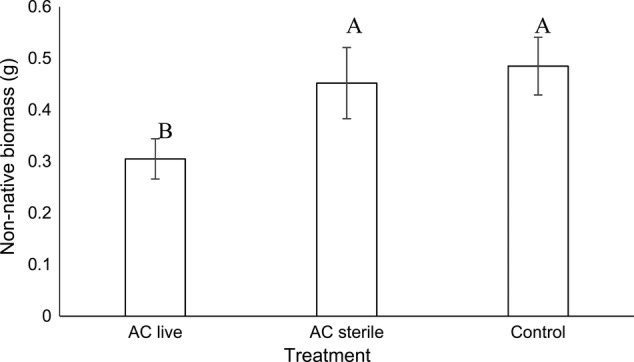
Non-native biomass (g) for individuals grown in monoculture in control, activated-carbon-treated (AC live) and activated-carbon-treated and sterilized (AC sterile) soils (mean ± SE). Letters denote differences among treatments at the α < 0.05 level.

The native : non-native aboveground biomass ratio was greater in AC live soil than AC sterile and control soils (*F*_2, 657_ = 3.29, *P* = 0.04; Fig. 4A). Native aboveground biomass in AC live soil was lower than native aboveground biomass in the control soil, while native aboveground biomass did not differ between AC sterile and AC live or control soil (*F*_2,657_ = 5.41, *P* < 0.01; Fig. 4B). Non-native aboveground biomass was lower in AC live soil than in AC sterile and control soils (*F*_2,657_ = 6.06, *P* < 0.01; Fig. 4B). This was also true for non-native species grown in monoculture (i.e. unsuccessful *B. sagittata* pairings; *F*_2,182_ = 5.18, *P* < 0.01; Fig. 5).

Native species growth differed among non-native pairings (*F*_4,657_ = 10.95, *P* < 0.01). Natives had the greatest biomass with *S. altissimum* and the least with *B. tectorum*. Non-native species growth similarly differed among native pairings (*F*_3,657_ = 4.48, *P* < 0.01). Non-natives had the greatest aboveground biomass with *L. sericeus* and the least with *K. macrantha*.

Belowground native biomass and non-native biomass showed no response to treatment (*F*_2,657_ = 1.83, *P* = 0.16 and *F*_2,657_ = 1.51, *P* = 0.22, respectively).

## Discussion

In both field and greenhouse experiments, AC decreased non-native plant growth and increased the relative abundance of native to non-native plants. At the end of the 3-year field experiment, this effect was evident at AC concentrations >400 g m^−2^ (Fig. 2D). In the greenhouse experiment, the fact that AC decreased non-native growth in monoculture pots (Fig. 5) indicated that AC effects occurred regardless of competition or allelopathy. The fact that non-native growth decreased only in live soils suggested that AC effects were microbially mediated (Figs 4 and 5). The fact that AC decreased non-native growth suggested that AC either increased negative plant–microbe interactions or decreased positive plant–microbe interactions. Because AC is known to indiscriminately bind the organic molecules that allow communication and the development of interactions among soil organisms, we suggest that the most likely explanation for the microbially mediated decrease in non-native plant growth in AC plots is that AC decreased the development of positive plant–microbe interactions that have recently been suggested to dominate plant–soil interactions (Kulmatiski *et al*. 2014). Microbial analyses suggested organisms that may play a role in these plant–microbe interactions.

Similar to previous research conducted on a smaller spatial scale (1 m × 1 m plots), we found that the addition of 1000 g m^−2^ of coal-based AC to larger plots (15 m × 15 m) using more real-world application techniques (i.e. tractor discing rather than hand mixing) increased the ratio of native to non-native plant species (Kulmatiski and Beard 2006; Kulmatiski 2011). Non-native species were the drivers of this community composition change (Figs 1 and 2). More specifically, non-native cover decreased from 24 % cover in seeded controls to 19 % cover in plots treated with 1000 g m^−2^ coal-based AC. This effect of AC was similar to the effect of adding native seed: adding native seed decreased non-native cover from 31 to 24 %.

**Figure 6.**
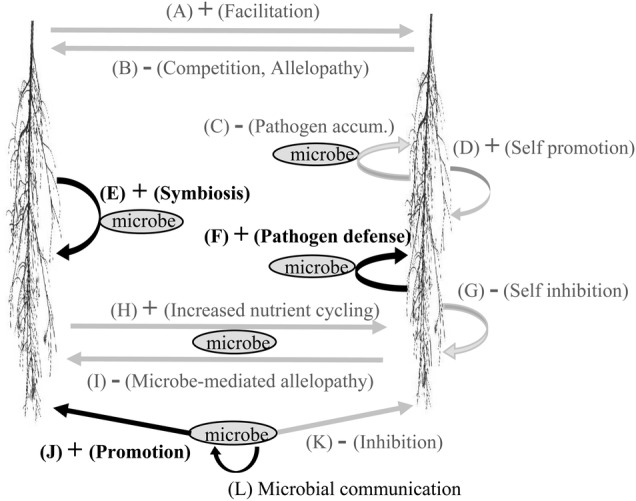
Potential plant–soil interactions that may be affected by activated carbon soil additions. Pathways supported by this research are shown in black. Pathways not supported by this research are shown in grey.

Greenhouse results provided insight into the potential mechanisms through which AC may increase the relative abundance of native plant growth. A number of positive and negative interactions occur among plant roots, soils and soil organisms that can affect plant growth (Fig. 6). Because AC binds organic molecules we suggest that AC is likely to decrease plant–soil communication and subsequent interactions. Therefore, we suggest that the decrease in plant growth caused by AC was more likely to be caused by decreases in positive plant–soil interactions (i.e. E, F and J in Fig. 6) than by increases in negative plant–soil interactions (i.e. B, C, G, I and K in Fig. 6). Results from our sterilization experiment in the greenhouse indicated that AC effects were only realized in live soils. Thus, AC did not appear to affect plant growth by suppressing facilitation or self-promotion (i.e. A and D in Fig. 6). The most likely potential mechanisms remaining are suppression of symbioses, suppression of pathogen defence and suppression of increased nutrient cycling (Hawkes *et al*. 2005). Results from our current and previous field experiments suggest that AC does not increase nutrient availability (Kulmatiski and Beard 2006). Therefore, we suggest that the most likely mechanisms through which AC affects plant growth is by suppressing (i) the development of symbiotic relationships between plants and soil organisms (Callaway and Aschehoug 2000; Mangla *et al*. 2008; Weißhuhn and Prati 2009; Wurst and Van Beersum 2009; Kulmatiski 2011) or (ii) the pathogen defence (Van der Putten 2003; Bais *et al.* 2004; Orians and Ward 2010; Doornbos *et al.* 2012). Future experiments would be needed to distinguish the importance of these two mechanisms.

Many experiments have been conducted testing allelopathic effects using AC (Lau *et al*. 2008; Wurst *et al*. 2010; Murrell *et al*. 2011); however, the results of our experiments suggest that for many of the dominant native and non-native species of the western USA, the effects of AC on plant–microbial interactions are more influential than its effects on allelopathy.

Results from microbial analyses are consistent with the idea that AC affects plant growth by changing microbial composition. Early in the spring, before plant growth, microbial communities in AC-treated soils (1000 g AC m^−2^) were more similar to undisturbed native soils than to control soils (0 g AC m^−2^). More specifically, early season AC soils and native soils were associated with an increase in an unidentified bacterium and Actinomycetales, and with a decrease in a Flavobacterium. However, late season, only native soils were associated with these OTUs. This suggested that AC created a soil microbial community that was similar to native plants, but that once non-native plants grow on these soils the microbial community returns to a composition more similar to that associated with non-native plants. Flavobacterium have been noted for their plant growth promoting activity (Soltani *et al*. 2010), so a decrease in Flavobacterium is consistent with a decrease in positive plant–microbe interactions. Actinomycetales have been found to affect fungal infection (Maier *et al.* 2004), so it is possible that AC treatments increased Actinomycetales, which either increased fungal pathogen infection or decreased mycorrhizal infection. These potential mechanisms are purely speculative, but help narrow down a long list of soil organisms to those that are more likely to have important effects on plant growth. Follow-up experiments testing the effects of these different organisms (i.e. tests of Koch's postulates) will be needed to test their effects on native and non-native plant growth.

Two other goals of this experiment were to test the effectiveness of (i) lower concentrations of AC and (ii) wood-based AC. We wanted to test the effectiveness of wood-based carbon because wood-based AC treatments would hold the benefit of fixing carbon into soils and may allow the development of on-site production. On-site carbon production could have the benefit of both removing woody materials that present a fire hazard while simultaneously fixing carbon in the soil. In both cases results were variable and therefore difficult to interpret. In the third year of the study, the native: non-native ratios were greater in 400, 700 and 1000 treatments suggesting that at least 400 g m^−2^ of AC are needed for native plant restoration. Results, however, were not consistent across years so further testing with different concentrations in different sites is recommended. Plant responses to wood-based AC were even more variable, with wood-based AC having a greater native : non-native ratio than coal-based AC in the first year and a lower native : non-native ratio than coal-based AC in the third year. It is difficult, therefore, to conclude whether or not wood-based AC is a suitable alternative to coal-based AC.

Native plant responses were not as strong in this study as in the previous smaller-scale field experiment (Kulmatiski and Beard 2006). This may have been because the larger-scale application techniques (i.e. tractor discing rather than hand mixing) used in the current study were likely to produce less consistent AC incorporation into the soil. Additionally, more than twice as much rain fell during the first spring of the large-scale experiment relative to the small-scale experiment (i.e. 21.5 versus 9.3 cm precipitation between March and May; Kulmatiski 2011). As a result, native plants established vigorously in control plots during the large-scale study, making it difficult for any treatments to increase native plant growth beyond seeding success. The fact that AC effects were observed in the greenhouse, small-scale study (Kulmatiski and Beard 2006) and the current large-scale study suggests that AC effects are consistent across a wide range of conditions.

There is rapidly growing interest in the use of biochar (Atkinson *et al*. 2010; Lehmann *et al*. 2011; Spokas *et al*. 2012). Biochar has typically been used to increase plant growth in agricultural settings. The positive effect of biochar has been attributed to the fact that biochar can increase N, release P, increase pH and release ethylene, among other mechanisms (Spokas *et al*. 2010; Lehmann *et al*. 2011; Biederman and Harpole 2013). Consistent with this ‘fertilization’ effect, biochar increases annual plant growth more than perennial plant growth (Biederman and Harpole 2013). Our results suggest that AC effects on plant growth are fundamentally different than biochar effects on plant growth. Our results suggest that AC decreases plant growth by suppressing beneficial plant–microbe interactions, and that this benefits native plants by suppressing the growth of weeds that rely on fast growth rates. Thus, we suggest that most biochar is unlikely to be weed suppressive unless it has a very high C : N ratio (e.g. >300 : 1) or it is applied at very high rates (i.e. >5 % by volume of soil) that can interfere with plant–microbe communication (Biederman and Harpole 2013).

It is necessary to consider the cost of AC application before considering broad use. Costs for tested AC concentrations are approximately: 100 g m^−2^: $454 ha^−1^, 400 g m^−2^: $1817 ha^−1^, 700 g m^−2^: $3180 ha^−1^, 1000 g m^−2^: $4543 ha^−1^ and 1000 w g m^−2^: $5618 ha^−1^. Due to its high cost, AC is not a practical large-scale restoration tool. However, it may be useful for targeted treatments, in relatively small sites, such as restoring abandoned oil pad sites. Because AC is eliciting community changes by decreasing positive microbial interactions with non-native species, it is likely more beneficial to further research and explore techniques that manipulate particular plant–microbe interactions (i.e. through inoculation).

## Conclusions

In a large-scale field application, AC increased native restoration even when applied in a year with a wet spring that resulted in vigorous native plant growth. Previous results from a small-scale experiment suggested that this effect may be greater in drier years. A greenhouse experiment suggested that AC decreases non-native plant growth by suppressing the development of positive plant–microbe interactions—not by suppressing allelopathy or nutrient cycling rates. Bacterial analyses suggested that Actinomycetales and Flavobacterium are good candidates for future testing of AC effects. Activated carbon application remains a blunt and costly restoration tool that may only have applications in high-value lands, but results demonstrate the potential for soil-based manipulations that can be used to guide plant community development.

## Sources of Funding

The work was funded by the USDA NRI (# 2010-85320-20402) and the USU Ecology Center. This research was supported by the Utah Agricultural Experiment Station, Utah State University and was approved as journal paper number 8701.

## Contributions by the Authors

N.E.N. analysed the data and led the writing. A.K. and K.H.B. conceived the experiments and oversaw the collection of field and lab data. J.M.N. oversaw the microbial component. A.K. was supported by a USDA AFRI grant.

## Conflicts of Interest Statement

None declared

## Supporting Information

The following supporting information is available in the online version of this article –

**Table S1.** Species grouping and classification for field experiment results.

Additional Information
