# Supplementary material for: Activated carbon decreases invasive plant growth by mediating plant–microbe interactions
Source: AoB Plants. 2014 Nov 10;7:plu072. doi: 10.1093/aobpla/plu072 (PMC4303759; doi:10.1093/aobpla/plu072)
Supplement: Additional Information [file supp_7_plu072_index.html]

Activated carbon decreases invasive plant growth by mediating plant–microbe interactions — Additional Information 

# Activated carbon decreases invasive plant growth by mediating plant–microbe interactions

## Additional Information

Additional Information

**Files in this Data Supplement:**

- Additional Information - docx file
