# Supplementary material for: Activated carbon decreases invasive plant growth by mediating plant–microbe interactions
Source: AoB Plants. 2014 Nov 10;7:plu072. doi: 10.1093/aobpla/plu072 (PMC4303759; doi:10.1093/aobpla/plu072)
Supplement: Additional Information [file supp_plu072_plu072supp.docx]

## Table 1. Species grouping and classification for field experiment results

| Species | Origin | Growth Habit | Duration | Categorization |
| --- | --- | --- | --- | --- |
| *Acroptilon repens*(L.) DC. | non-native | forb | perennial | undesirable |
| *Agoseris glauca*(Pursh) Raf. | native | forb | perennial | desirable |
| *Agropyron cristatum*(L.) Gaertn. | non-native | grass | perennial | undesirable |
| *Amaranthus albus*L. | non-native | forb | annual | undesirable |
| *Amsinckia menziesii*(Lehm.) A. Nelson & J.F. Macbr. | native | forb | annual | undesirable |
| *Artemisia tridentata*Nutt. | native | shrub | perennial | desirable |
| *Balsamorhiza sagittata*(Pursh) Nutt. | native | forb | annual | desirable |
| *Borago officinalis*L. | non-native | forb | annual | undesirable |
| *Bromus inermis*Leyss. | non-native | grass | perennial | undesirable |
| *Bromus tectorum*L. | non-native | grass | annual | undesirable |
| *Buglossoides arvensis*(L.) I.M. Johnst. | non-native | forb | annual | undesirable |
| *Cardaria draba*(L.) Desv. | non-native | forb | perennial | undesirable |
| *Centaurea diffusa*Lam. | non-native | forb | perennial | undesirable |
| *Chenopodium album*L. | native | forb | annual | undesirable |
| *Chorispora tenella*(Pall.) DC. | non-native | forb | annual | undesirable |
| *Collinsia parviflora*Lindl. | native | forb | annual | undesirable |
| *Collomia grandiflora*Douglas ex Lindl. | native | forb | annual | desirable |
| *Convolvulus arvensis*L. | non-native | forb | perennial | undesirable |
| *Festuca idahoensis*Elmer | native | grass | perennial | desirable |
| *Hesperostipa comata*(Trin. & Rupr.) Barkworth | native | grass | perennial | desirable |
| *Koeleria cristata* Schult. | native | grass | perennial | desirable |
| *Lactuca serriola*L. | non-native | grass | annual | undesirable |
| *Leymus cinereus*(Scribn. & Merr.) Á. Löve | native | grass | perennial | desirable |
| *Lomatium spp.* | native | forb | perennial | desirable |
| *Lomatium dissectum*(Nutt.) Mathias & Constance | native | forb | perennial | desirable |
| *Lupinus sericeus*Pursh | native | forb | perennial | desirable |
| *Madia citriodora*Greene | native | forb | annual | undesirable |
| *Medicago sativa*L. | non-native | forb | perennial | undesirable |
| *Microsteris gracilis*(Hook.) Greene var. *humilior*(Hook.) Cronquist | native | forb | annual | undesirable |
| *Phacelia linearis*(Pursh) Holz. | native | forb | annual | undesirable |
| *Poa bulbosa*L. | non-native | grass | perennial | undesirable |
| *Polygonum douglasii* Greene | native | forb | annual | undesirable |
| *Pseudoroegneria spicata*(Pursh) Á. Löve | native | grass | perennial | desirable |
| *Purshia tridentata*(Pursh) DC. | native | shrub | perennial | desirable |
| *Sisymbrium altissimum* L. | non-native | forb | annual | undesirable |
| *Taraxacum*F.H. Wigg. | non-native | forb | perennial | undesirable |
| *Thlaspi arvense* L. | non-native | forb | perennial | undesirable |
| *Tragopogon dubius*Scop. | non-native | forb | annual | undesirable |
